# Plasma proteins and psoriatic arthritis: a proteome-wide Mendelian randomization study

**DOI:** 10.3389/fimmu.2024.1417564

**Published:** 2024-07-04

**Authors:** Heran Zhao, Yi Zhou, Ziyan Wang, Xuan Zhang, Leilei Chen, Zhinan Hong

**Affiliations:** ^1^ Department of Orthopaedics, The Third Affiliated Hospital of Guangzhou University of Chinese Medicine, Guangzhou, China; ^2^ The Third Clinical College, Guangzhou University of Chinese Medicine, Guangzhou, China; ^3^ Graduate School, Nanjing University of Chinese Medicine, Nanjing, China; ^4^ College of Orthopedics and Traumatology, Guangxi University of Chinese Medicine, Nanning, China

**Keywords:** psoriatic arthritis, plasma protein, Mendelian randomization, drug target, single nucleotide polymorphism

## Abstract

**Background:**

Previous epidemiological studies have identified a correlation between serum protein levels and Psoriatic Arthritis (PsA). However, the precise nature of this relationship remains uncertain. Therefore, our objective was to assess whether circulating levels of 2,923 plasma proteins are associated with the risk of PsA, utilizing the Mendelian randomization (MR) approach.

**Methods:**

Two-sample MR analysis was performed to assess the causal impact of proteins on PsA risk. Exposure data for plasma proteins were sourced from a genome-wide association study (GWAS) conducted within the UK Biobank Pharma Proteomics Project, which encompassed 2,923 unique plasma proteins. The outcome data for PsA were sourced from the FinnGen study, a large-scale genomics initiative, comprising 3,537 cases and 262,844 controls. Additionally, colocalization analysis, Phenome-wide MR analysis, and candidate drug prediction were employed to identify potential causal circulating proteins and novel drug targets.

**Results:**

We thoroughly assessed the association between 1,837 plasma proteins and PsA risk, identifying seven proteins associated with PsA risk. An inverse association of Interleukin-10 (IL-10) with PsA risk was observed [odds ratio (OR)=0.45, 95% confidence interval (CI), 0.28 to 0.70, *P*_FDR_=0.072]. Additionally, Apolipoprotein F (APOF) has a positive effect on PsA risk (OR=2.08, 95% CI, 1.51 to 2.86, *P*_FDR_=0.005). Subsequently, we found strong evidence indicating that IL-10 and APOF were colocalized with PsA associations (PP.H4 = 0.834 for IL-10 and PP.H4 = 0.900 for APOF). Phenome-wide association analysis suggested that these two proteins may have dual effects on other clinical traits (*P*_FDR_<0.1)

**Conclusion:**

This study identified 7 plasma proteins associated with PsA risk, particularly IL-10 and APOF, which offer new insights into its etiology. Further studies are needed to assess the utility and effectiveness of these candidate proteins.

## 1 Introduction

Psoriatic arthritis (PsA) is a prevalent chronic inflammatory disease characterized by a propensity to affect the peripheral and axial skeleton, resulting in musculoskeletal injuries, and associated with psoriasis (1). Recent studies indicate that PsA, afflicting up to 30% of psoriasis patients, has a prevalence of 6-25 cases per 10,000 individuals in the American population (2). Although PsA primarily manifests in joint pathology, emerging evidence suggests that patients with PsA are at higher risk for cardiovascular diseases, respiratory disorders, and malignancies (3, 4). Moreover, PsA significantly impacts patients’ physical and mental health and imposes substantial financial burdens. For instance, individuals with PsA often suffer from absenteeism and decreased work productivity (5). Given the substantial disease burden associated with PsA and the existing limitations in its treatment options, delving into the potential mechanisms and therapeutic targets of PsA holds significant societal value.

Proteins present in the bloodstream, whether stemming from cellular leakage or active secretion, provide important information about the health of organisms and constitute a primary source of biomarkers and therapeutic targets (6, 7). Prior investigations have established associations between protein levels and PsA, suggesting their potential significance in PsA management (8, 9). For instance, a study involving 27 PsA patients revealed a strong correlation between IL-23 transcriptomic/protein expression and high-grade synovitis in PsA cases (10). Furthermore, a multicenter retrospective study comparing 221 PsA patients highlighted the potential symptom-alleviating effects of IL-17 in moderate-to-severe cases (11). However, the potential to uncover causal associations between protein markers and the risk of PsA is often limited by several factors, including small sample sizes, observational designs, restricted types of proteins, or the narrow range of methods used.

Mendelian randomization (MR) stands as a well-established method in genetic epidemiology, serving as instrumental variables (IVs) within non-experimental designs to infer causality between modifiable exposures and disease outcomes (12). This method leverages the presumed random inheritance of genetic variants, unaffected by disease processes, thereby mitigating confounding factors and reversing causation biases (12). Recently, Proteome-wide MR analysis, leveraging genome-wide association study (GWAS) findings, has enabled a more comprehensive understanding of genetic determinants while uncovering potential therapeutic targets associated with various complex diseases, particularly those influenced by circulating proteins (13, 14). Therefore, together with the accessibility of the blood plasma database, we combined a two-sample MR analysis, colocalization analysis, and Phenome-wide MR (Phe-MR) analysis to identify the potentially causal plasma proteins and novel drug targets in PsA.

## 2 Methods

### 2.1 Study design

**Figure 1:**
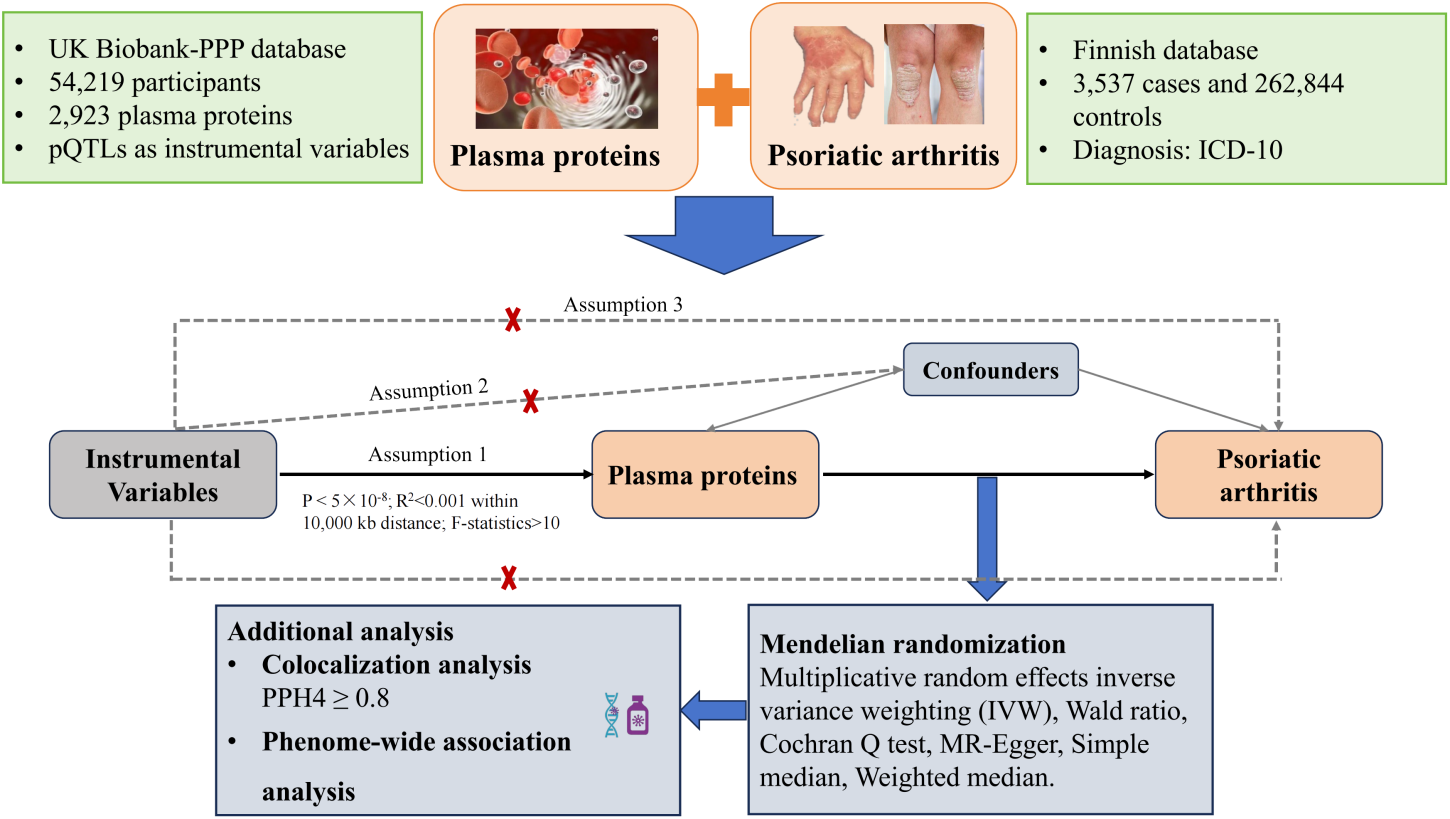
The study design of the association between plasm protein and risk of psoriatic arthritis.

This study utilized pQTL data sourced from an extensive proteomic investigation and scrutinized their connections with PsA through MR analysis. Furthermore, Bayesian colocalization was employed to validate the relationships between 2,923 protein biomarkers with PsA risk. Subsequently, Phe-MR analyses were performed to assess the druggability of identified protein biomarkers and prioritize prospective treatment targets (**Figure 1**).

### 2.2 Exposure data sources for plasma proteins

Index single nucleotide polymorphisms (SNP) associated with the levels of plasma proteins were sourced from the UK Biobank Pharma Proteomics Project (UKB-PPP) (15). UKB-PPP performed proteomic profiling on blood plasma samples from 54,219 UK Biobank participants using the Olink platform and collected 2,923 protein data (https://www.synapse.org/#!Synapse:syn51365301) (15). In the MR study, we chose pQTLs as IVs, using the subsequent selection criteria: 1) SNP within ± 10000 kb of the gene region; 2) Genome-wide significance (*P <*5×10^-8^) for selecting highly correlated SNPs with 2,923 plasma proteins; 3) A threshold of 0.001 for the LD parameter (r^2^) and distance of 10,000 kb to ensure independent SNPs and minimize LD impact; 4) Exclusion of weak IVs with an F-value greater than 10 (16). We used the TwoSampleMR package, function “harmonize data” to carry out the SNP harmonization.

### 2.3 Outcome data sources for psoriatic arthritis

The associations of protein-associated SNPs with PsA were scored from the FinnGen study (https://r10.finngen.fi/). The FinnGen study examined more than 500,000 samples from the Finnish biobank and linked genetic information with health data (17). We used the recent public data on PsA from the FinnGen R10, which comprised 3,537 cases and 262,844 controls in this study (17).

### 2.4 MR analysis

In this MR analysis, we employed multiplicative random effects inverse variance weighting (IVW), MR-Egger, Wald ratio, and weighted median to evaluate protein-PsA causality. For each selected SNP, we calculated the Wald ratio to estimate the causal effect for each instrumental SNP, followed by IVW meta-analysis for each Wald ratio (18). MR-Egger addresses certain violations of the standard IV assumptions, and its intercept offers a robust causal effect estimate after horizontal pleiotropy adjusting (19). The weighted median method provides an unbiased causal effect estimator when up to half of the IVs are invalid, demonstrating lower causal effect detection than the IVW method but with reduced bias and a lower type I error rate (20). In this study, the IVW method was employed as the primary approach, which is analogous to conducting a weighted linear regression of the PsA association on the protein associations (21). Heterogeneity among IVs was assessed using the Cochran Q test, with a heterogeneity Q *P* > 0.05 indicating no significant heterogeneity (22).

### 2.5 Colocalization analysis

Colocalization analysis can judge whether two traits share causal variants within a single region (23), testing five hypotheses: (i) H_0_ indicates no association with either trait; (ii) H_1_ suggests association with trait 1, not trait 2; (iii) H_2_ indicates association with trait 2, not trait 1; (iv) H_3_ implies association with both traits via two independent SNPs; (v) H_4_ association with both traits through a shared SNP. A higher posterior probability (PP) of the H4 (PP.H4) provides evidence for the significant MR findings. In this study, PP.H4 > 0.8 was considered supportive evidence of colocalization (16).

### 2.6 Phenome-wide association analysis

Phenome-wide association studies have been used in pharmaceutical development by clarifying mechanisms of action, detecting alternative uses or predicting adverse drug events (ADEs) (24). To further evaluate the potential drug targets and possible ADEs, Phe-MR analysis was performed for the significant proteins associated with PsA to include phenotypes for a wide range of diseases. To avoid data duplication, we used the FinnGen R10 cohort (https://r10.finngen.fi/), including 412,181 participants. The disease outcomes were defined based on “PheCodes” for systematic genetic analysis of the disease traits (25). Diseases with less than 50 cases were excluded from the study, leaving 2,408 diseases for Phe-MR analyses.

In this study, all analyses were performed using the ‘TwoSampleMR’ and ‘coloc’ packages in R version 4.1.2. We utilized the False Discovery Rate (FDR) method to adjust P-values due to multiple calculations, with a PFDR < 0.10 considered statistically significant (26).

## 3 Results

### 3.1 Proteins effect on PsA

**Figure 2:**
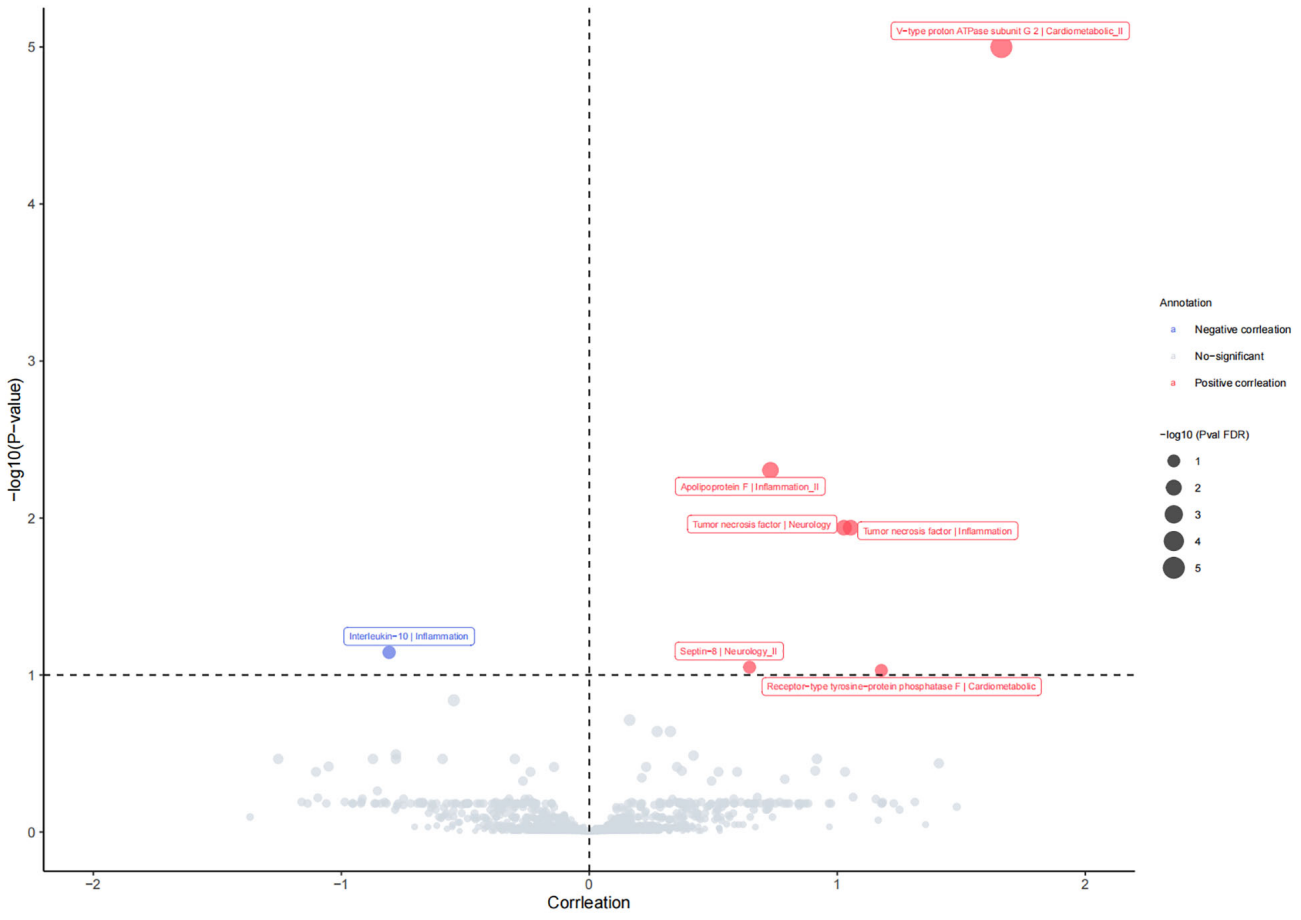
The causal relationship between plasm protein and risk of psoriatic arthritis in the MR analysis. *P*_FDR_ <0.1 was considered as significant association.

A total of 1,837 plasma proteins were ultimately included in the MR analysis. The F-values for selected IVs ranged from 14.67 to 20740.35, indicating the absence of weak instrumental bias affecting causal associations. Further details for selected SNP information are available in **Supplementary Table 1**. We comprehensively evaluated the impact of these 1,837 proteins on PsA using the IVW or Wald ratio methods. Our analysis revealed that seven plasma proteins are associated with the incidence of PsA (*P*_FDR_ <0.1; **Figure 2**). As indicated in **Supplementary Table 1**, two inflammatory proteins, Tumor necrosis factor (TNF) and Apolipoprotein F (APOF), exhibited an association with an increased risk of PsA, whereas Interleukin-10 (IL-10) showed a negative association with PsA risk. Additionally, there were positive associations observed between two proteins related to cardiometabolic factors and the risk of PsA [V-type proton ATPase subunit G 2 (ATP6V1G2), OR=5.27, 95% CI, 3.80 to 7.30, *P*_FDR_ =1.99×10^-20^; Receptor-type tyrosine-protein phosphatase F (PTPRF), OR=3.25, 95% CI, 1.63 to 6.48, *P*_FDR_ = 0.093]. Moreover, MR analysis revealed that genetic predisposition for TNF and Septin-8 (SEPTIN8), both related to neurology, exhibited positive associations with PsA risk (TNF, OR=2.79, 95% CI, 1.70 to 4.59, *P*_FDR_ =0.012; SEPTIN8, OR=1.91, 95% CI, 1.31 to 2.77, *P*_FDR_ = 0.089). More details for the association of proteins with PsA are presented in **Supplementary Tables 2**–**4**.

### 3.2 Colocalization analysis

Gene colocalization analysis for 7 proteins (spanning the upstream and downstream regions) in this study indicated that IL-10 may share a causal variant in the region (PP.H4 = 0.834). Additionally, APOF was colocalized with PsA associations with strong evidence (PP.H4 for APOF = 0.900). However, no common causal variant was observed among the other five proteins and PsA in these regions (all PP.H4<0.8, **Supplementary Table 5**).

### 3.3 Phenome-wide MR analysis

**Figure 3:**
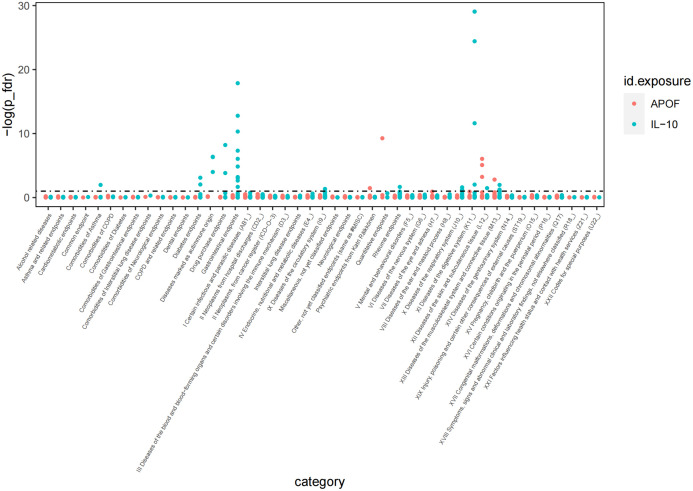
The phenome-wide MR analysis for IL-10 and APOF.

To comprehensively elucidate the impact of these proteins such as IL-10 and APOF linked to PsA on other clinical traits, we screened over 2,000 traits from the Finnish GWAS (version 10) and performed a Phe-MR association analysis. Our findings revealed that IL-10 was linked to 32 traits, while APOF was associated with six traits, respectively (*P*_FDR_<0.1; **Figure 3**). Particularly noteworthy was the inverse association between IL-10 and 28 clinical traits, suggesting a potential protective effect of IL-10 on these clinical traits. However, IL-10 was found to increase the risk of nasal polyps, and type 1 diabetes, indicative of eosinophilic asthma (**Supplementary Table 6**). On the other hand, APOF showed positive associations with three traits, including papulosquamous disorders, psoriasis vulgaris, and inverse associations with two traits (**Supplementary Table 6**). Notably, we observed that APOF increased PsA risk by 60% (OR=1.69, 95% CI, 1.40 to 2.02, *P*_FDR_ <0.001), while IL-10 reduced PsA by 40% risk (OR=0.60, 95% CI, 0.46 to 0.79, *P*_FDR_=0.034).

## 4 Discussion

To our knowledge, this study is the first to employ an integrated approach combining MR, colocalization, and Phe-MR analysis to explore the causal effects of 1,837 plasma proteins from UKB-PPP on PsA risk based. We identify seven proteins associated with PsA, demonstrating a vital role in the pathogenesis of it. Among them, five proteins (ATP6V1G2, APOF, TNF, SEPTIN8, and PTPRF) significantly increase the risk of PsA, while only IL10 is associated with a reduced risk. Colocalization analysis confirmed that APOF and IL-10 would have more potential to become drug targets for PsA. Phe-MR MR test was performed to find some beneficial or adverse drug events (ADEs) with APOF and IL-10.

APOF, known as Apolipoprotein F, is a minor apolipoprotein present in human plasma that primarily binds to lipoproteins involved in cholesterol transportation and esterification (27). Previous observational studies have provided evidence similar to our analysis. For instance, a US-based cohort study involving 95,540 female participants showed that hypercholesterolemia increased PsA risks by 58% (HR=1.58, 95% CI, 1.13 to 2.23) (28). Similarly, other studies have reported elevated cholesterol levels in PsA patients irrespective of the presence of obesity (29, 30). Hypercholesterolemia alters the microcirculation’s phenotype from anti-inflammatory to pro-inflammatory, possibly due to elevated reactive oxygen species levels and reduced nitric oxide bioavailability (31, 32). Additionally, hypercholesterolemia can induce microvascular inflammation involving the immune system. Evidence indicates that high cholesterol levels activate both innate and adaptive immune systems in postcapillary venules, with T-lymphocytes potentially being among the first cell types activated by hypercholesterolemia (33, 34). Our MR findings provide more reliable evidence for causal inference than observational studies with possible reverse causation and relatively small sample sizes.

A crucial anti-inflammatory mediator, IL-10 protects the body from overreacting to infections and bacteria (35). Our results found that lower IL-10 protein levels are associated with a reduced PsA risk, which aligns with other studies. A study observed that had an increase in the number of IL-10-producing B10 cells in 20 patients with PsA after treatment with apremilast (36). This change correlated with clinical improvement in psoriatic skin and joint symptoms, suggesting a potential for IL-10 to ameliorate PsA symptoms (36). Similarly, a case-control study of 133 individuals showed that PsA patients had lower levels of IL-10 compared to healthy controls, with IL-10 levels negatively correlated with PsA severity (37). IL-10 may impede the effective development of T cell responses by altering the local cytokine microenvironment restricting antigen delivery, and potentially inhibiting PsA formation (35). Additionally, IL-10 decreases the beginning of psoriasis by regulating regulatory B cells, which is another way in which it performs its anti-inflammatory role (37). Based on these studies, IL-10 emerges as a promising therapeutic target for PsA, though further experimental animal studies are needed.

TNF is a pivotal immune response cytokine, closely associated with the initiation and progression of various inflammatory and autoimmune diseases (38). Extensive research has suggested the critical role of TNF in chronic inflammatory conditions. Therapeutics that specifically target TNF (adalimumab, golimumab, and etanercept) have demonstrated notable efficacy in managing chronic inflammatory and autoimmune conditions (38–40). Serum TNF-α levels PsA severity were found to be correlated in an observational study involving 213 individuals (41). Similarly, a multicenter observational study conducted across four Italian centers concluded that TNF-α blockers effectively achieved low disease activity and were safe in PsA patients (42). This effectiveness may be attributed to TNF-α blockers preventing joint destruction by inhibiting TNF’s promotion of cartilage- and bone-degrading enzyme (43). Additionally, TNF is critical in erosion development by promoting receptor activator of nuclear factor-κB (NF-κB) ligand expression in the synovium, essential for osteoclast differentiation (44). Our study provides compelling evidence suggesting TNF’s potential role in PsA development.

ATP6V1G2 is a gene encoding a component of vacuolar ATPase (V-ATPase), which plays a key role in the acidification of intracellular compartments in eukaryotic cells (45). Although the association between ATP6V1G2 and PsA risk has not been extensively explored, a potential positive correlation can be postulated based on available indirect evidence. ATP6V1G2 is situated in the HLA region, akin to the psoriasis susceptibility region containing HLA-C, such as TNF (46). Moreover, V-ATPase has been implicated in tumor invasion, metastasis, and alterations in the immune microenvironment, necessitating further investigations to elucidate complex connections (45). SEPTIN8, a family of 13 GTP-binding proteins involved in regulating axon and dendrite formation, growth, stability, synaptic plasticity, and autophagy (47). However, studies evaluating the relationship between SEPTIN8 and PsA risk have been limited. Only a few studies have associated SEPTIN8 with certain neurological disorders (48, 49). Initially identified though its role in leukemia research, PTPRF regulates cell proliferation and differentiation by acting on downstream substrates, akin to the function of many other phosphatases (50, 51). Similar to ATP6V1G2 and SEPTIN8, limited research has reported on the association between PTPRF and PsA risk. However, our MR results suggest an unexpected positive association between ATP6V1G2, SEPTIN8, PTPRF, and PsA risk. This suggests a potentially more complex relationship than expected, necessitating further studies to clarify this contradiction and to comprehend the roles of the three proteins in the development of PsA.

The primary strength of our study is the two-sample MR analysis utilizing data from a large-scale GWAS in the UKB-PPP and PsA from the FinnGen study. Our findings shed light on potential plasma protein targets for PsA drug development. Furthermore, our study design incorporates several methodological strategies to minimize potential confounders and biases, including MR to mitigate reverse causality, prioritization of cis-pQTLs over trans-pQTLs and eQTLs to enhance evidence credibility, and gene colocalization analyses to bolster statistical robustness. However, this study also has several limitations. Firstly, the cohorts utilized in our MR analysis comprised individuals of European descent, limiting the generalizability to non-European populations. Besides, the MR analysis was conducted using summary-level data, preventing matching for factors like sex and age, which may have influenced our findings. Secondly, due to the limited number of GWAS studies on PsA and constraints in study cohorts, we were unable to conduct a replication study, necessitating further investigations to validate our results. Moreover, we could not identify significant plasma proteins associated with psoriasis limited to its cutaneous manifestations (without rheumatic involvement), owing to the constraints of available summary data. Finally, while this study evaluated the role of plasma proteins in PsA, we have no information on the levels of relevant proteins in other tissues. Evaluating protein levels in other tissues may yield further insights into PsA pathogenesis. While our study provides valuable insights into potential plasma protein targets for PsA drug development, further research is warranted to address the limitations and deepen our understanding of PsA pathophysiology, particularly in diverse populations and across various tissue types.

## 5 Conclusion

In conclusion, this study has identified 7 plasma proteins linked to the risk of PsA, particularly IL-10 and APOF, which identify novel therapeutic targets. Further research is required to validate the association between these candidate plasma proteins with PsA risk.

## Data availability statement

The original contributions presented in the study are included in the article/**Supplementary Material**. Further inquiries can be directed to the corresponding authors.

## Ethics statement

Ethical approval was not required for the study involving humans in accordance with the local legislation and institutional requirements. Written informed consent to participate in this study was not required from the participants or the participants’ legal guardians/next of kin in accordance with the national legislation and the institutional requirements.

## Author contributions

HZ: Visualization, Software, Methodology, Investigation, Formal analysis, Writing – original draft. YZ: Visualization, Software, Methodology, Investigation, Formal analysis, Writing – original draft. ZW: Software, Project administration, Data curation, Writing – original draft. XZ: Resources, Funding acquisition, Writing – review & editing. LC: Supervision, Resources, Project administration, Funding acquisition, Writing – review & editing. ZH: Supervision, Resources, Project administration, Funding acquisition, Writing – review & editing.
